# The rice terpene synthase gene *OsTPS19* functions as an (*S*)‐limonene synthase *in planta,* and its overexpression leads to enhanced resistance to the blast fungus *Magnaporthe oryzae*

**DOI:** 10.1111/pbi.12914

**Published:** 2018-04-06

**Authors:** Xujun Chen, Hao Chen, Joshua S. Yuan, Tobias G. Köllner, Yuying Chen, Yufen Guo, Xiaofeng Zhuang, Xinlu Chen, Yong‐jun Zhang, Jianyu Fu, Andreas Nebenführ, Zejian Guo, Feng Chen

**Affiliations:** ^1^ Key Laboratory of Plant Pathology Department of Plant Pathology China Agricultural University Beijing China; ^2^ Department of Plant Sciences University of Tennessee Knoxville TN USA; ^3^ Department of Plant Pathology and Microbiology Texas A&M University College Station TX USA; ^4^ Max Planck Institute for Chemical Ecology Jena Germany; ^5^ State Key Laboratory for Biology of Plant Diseases and Insect Pests Institute of Plant Protection Chinese Academy of Agricultural Sciences Beijing China; ^6^ Tea Research Institute Chinese Academy of Agricultural Sciences Hangzhou China; ^7^ Department of Biochemistry and Cellular and Molecular Biology University of Tennessee Knoxville TN USA

**Keywords:** *Oryza sativa*, limonene synthase, disease resistance

## Abstract

Rice blast disease, caused by the fungus *Magnaporthe oryzae*, is the most devastating disease of rice. In our ongoing characterization of the defence mechanisms of rice plants against *M. oryzae*, a terpene synthase gene *OsTPS19* was identified as a candidate defence gene. Here, we report the functional characterization of *OsTPS19*, which is up‐regulated by *M. oryzae* infection. Overexpression of *OsTPS19* in rice plants enhanced resistance against *M. oryzae*, while *OsTPS19* RNAi lines were more susceptible to the pathogen. Metabolic analysis revealed that the production of a monoterpene (*S*)‐limonene was increased and decreased in *OsTPS19* overexpression and RNAi lines, respectively, suggesting that OsTPS19 functions as a limonene synthase *in planta*. This notion was further supported by *in vitro* enzyme assays with recombinant OsTPS19, in which OsTPS19 had both sesquiterpene activity and monoterpene synthase activity, with limonene as a major product. Furthermore, in a subcellular localization experiment, OsTPS19 was localized in plastids. OsTPS19 has a highly homologous paralog, OsTPS20, which likely resulted from a recent gene duplication event. We found that the variation in OsTPS19 and OsTPS20 enzyme activities was determined by a single amino acid in the active site cavity. The expression of *OsTPS20* was not affected by *M. oryzae* infection. This indicates functional divergence of OsTPS19 and OsTPS20. Lastly, (*S*)‐limonene inhibited the germination of *M. oryzae* spores *in vitro*. OsTPS19 was determined to function as an (*S*)‐limonene synthase in rice and plays a role in defence against *M. oryzae,* at least partly, by inhibiting spore germination.

## Introduction

Rice blast disease caused by *Magnaporthe oryzae*, one of the top 10 fungal pathogens (Dean *et al*., 2012), is the most devastating rice disease, causing severe loss of production. As a model system for molecular studies, rice and *M. oryzae* have been investigated for pathogenicity, host resistance and their interactions. The basal resistance triggered by pathogen‐associated molecular patterns and specific effector‐triggered immunity is associated with a similar network and activate innate immune responses, including global transcriptional reprogramming (Zhang and Zhou, 2010). Rice defences, which are activated by blast resistance (called *Pi*) genes, often break down in practical applications because the pathogen effectors evolve rapidly to evade recognition by the corresponding *Pi* genes. Therefore, efforts have been undertaken to dissect the molecular processes as well as metabolites related to plant defences and provide useful resources for breeding of durable disease resistance. Recently, a natural allele of a C_2_H_2_‐type transcription factor in the rice cultivar Digu was found to confer nonrace‐specific resistance against blast fungi (Li *et al*., 2017). In addition to innate immune responses, plants also established an inducible immune response system, including systemic acquired resistance (SAR). Plant‐synthesized metabolites, such as salicylic acid (SA), pipecolic acid, azelaic acid and glycerol‐3‐phosphate, function as signal molecules in SAR (Fu and Dong, 2013). It was recently reported that volatile monoterpenes, particularly pinenes, promote SAR within and between *Arabidopsis* plants (Riedlmeier *et al*., 2017).

Upon pathogen infection, plants produce low‐molecular weight compounds with antimicrobial activities, such as phenolics, terpenoids and glucosinolates. Most of them are categorized as secondary metabolites. In the case of blast fungal attack, rice plants accumulate several types of diterpenoids and the flavonoid sakuranetin (methylated naringenin) as phytoalexins (Ahuja *et al*., 2012). To develop strategies to protect plants from biotic stresses, efforts have also been devoted to regulate the accumulation of defence chemicals, especially by identifying the genes responsible for metabolite biosynthesis. In rice, many such genes have been identified, such as the terpene synthase genes and SA methyltransferase genes involved in the production of insect‐induced volatiles (Yuan *et al*., 2008; Zhao *et al*., 2010). An increase in neomenthol and menthol contents confers pepper and *Arabidopsis* resistance against bacterial and fungal pathogens (Choi *et al*., 2008).

A number of terpene synthases (TPSs) from a range of plant species have been characterized, and some of them have been used for metabolic manipulation (Aharoni *et al*., 2006; Yu and Utsumi, 2009). Ectopic expression of lemon α‐zingiberene synthase, a sesquiterpene synthase gene, increased the accumulation of α‐zingiberene and other sesquiterpenes and monoterpenes in tomato (Davidovich‐Rikanati *et al*., 2008). (*E*)‐β‐caryophyllene in rice and maize has been shown to play a role in the attraction of parasitoid wasps of *Anagrus nilaparvatae* or entomopathogenic nematodes (Cheng *et al*., 2007; Degenhardt *et al*., 2009). Genetic modification has also been applied to change the composition of essential oils, primarily composed of monoterpenes and sesquiterpenes. The compound mixtures are used as fragrances, flavours and chemopreventive agents to protect plants from pathogen infection (Gershenzon and Dudareva, 2007). For example, overexpression of a limonene synthase gene from spearmint (*Mentha spicata*) altered the monoterpene composition of developing leaves of transgenic spike lavender (Muñoz‐Bertomeu *et al*., 2008). In addition, heterologous expression of monoterpene synthase genes from lemon altered the fragrance of tobacco plants (Lücker *et al*., 2004). The monocyclic monoterpene limonene has been found to be released from rice plants constitutively, and its emission is enhanced under abiotic and biotic stress conditions (Lee *et al*., 2015, 2016; Lou *et al*., 2006; Obara *et al*., 2002; Yuan *et al*., 2008).

Two recently duplicated terpene synthase genes, *OsTPS19* (*Os04g27190*) and *OsTPS20* (*Os04g27340*), were ascribed different functions as reported by Taniguchi *et al*. (2014) and Lee *et al*. (2015, 2016), respectively*. OsTPS20* was found to be induced by oxidative stress or the bacterial pathogen *Xanthomonas oryzae* pv. *oryzae* (*Xoo*) (Lee *et al*., 2015, 2016). The recombinant OsTPS20 protein has been shown to produce many monoterpenes including (*S*)‐limonene (Lee *et al*., 2015). In another report, *OsTPS19* was characterized as a sesquiterpene β‐elemene synthase gene (Taniguchi *et al*., 2014). In our previous studies, *OsTPS19* was induced remarkably in rice plants by fall armyworm (*Spodoptera frugiperda,* FAW) infestation (Yuan *et al*., 2008). Accumulated data indicate that transcription factors, such as WRKYs family members, play important roles in regulation of primary and secondary metabolic pathways (Akagi *et al*., 2014; Han *et al*., 2014; Liang *et al*., 2017; Xu *et al*., 2004). In searching for potential target genes of the transcription factor OsWRKY89, *OsTPS19* was found to be up‐regulated in *OsWRKY89* overexpression lines, which enhanced resistance to the rice blast fungus *M. oryzae* and *S. furcifera* (Wang *et al*., 2007). In this article, we are interested in identifying genes of secondary metabolism that are involved in *OsWRKY89*‐mediated rice defence again *M. oryzae*. Here, we provide biochemical, subcellular localization and transgenic evidence to support that *OsTPS19* functions as a limonene synthase *in planta*. Overexpression of *OsTPS19* in rice led to enhance the resistance against *M. oryzae*.

## Results

### Induced expression of *OsTPS19* in relation to rice defence against *M. oryzae* infection

**Figure 1:**
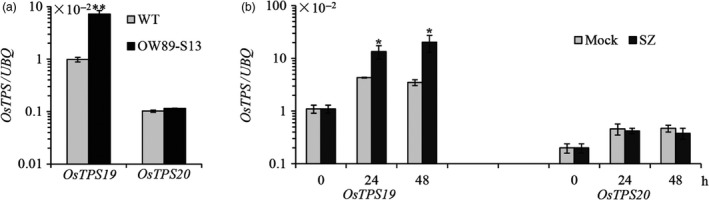
Expression of *OsTPS19* was increased in the *OsWRKY89* overexpression line and was induced by the rice blast fungus. (a) Expression of *OsTPS19* and *OsTPS20* in the *OsWRKY89* overexpression rice line (OW89‐S13). (b) Induction of *OsTPS19* and *OsTPS20* transcription by inoculation of the virulent rice blast fungus *M. oryzae* SZ (SZ). The transcription level of each gene was normalized with rice *ubiquitin* gene (*UBQ*). WT represents the wild‐type control plants; Mock represents the control treatments. Values presented are the means ± SD of three separate analyses for each RNA template; similar results were obtained from each duplicate. Asterisks indicate statistically significant differences (Student's *t* test; *P < 0.05 and **P < 0.01).

Transcription of *OsTPS19* was increased in transcription factor *OsWRKY89* overexpression plants, according to the microarray analysis data. To confirm this result, specific primers were designed to distinguish *OsTPS19* and *OsTPS20*, two close homologs with an amino acid identity of 95.5% (nucleotide identity of 97.3% in the open reading frame region) on chromosome 4. As shown in Figure 1a, the level of *OsTPS19* mRNA accumulation was markedly increased in the *OsWRKY89* overexpression plants (OW89‐S13, Figure S1), whereas the transcription level of *OsTPS20* did not change significantly.

Induction of *OsTPS19* and *OsTPS20* was analysed in three‐week‐old rice seedlings inoculated with *M. oryzae*, a causal agent of rice blast fungus. As shown in Figure 1b, only *OsTPS19* transcription was induced by a virulent *M. oryzae* SZ strain in comparison with the mock treatment. As there was a significant induction of *OsTPS19* in the mock treatment (Figure 1b), we speculate that expression of *OsTPS19* was related to diurnal rhythms because some volatile terpenoids are released in a fluctuating pattern (Cheng *et al*., 2007; Yazaki *et al*., 2017). *OsTPS19* transcription showed an oscillating pattern with the shifting of dark/light periods, that is an increase during the dark period, reaching a peak at the switching point of the dark/light periods (Figure S2). The induction pattern of *OsTPS19* expression gave an explanation for increase its transcription in the mock treatment of pathogen infection in which the rice seedlings were kept in the dark for 24 h under high humidity (Figure 1b).

### Altered expression of *OsTPS19* led to changes in rice resistance against *M. oryzae*

**Figure 2:**
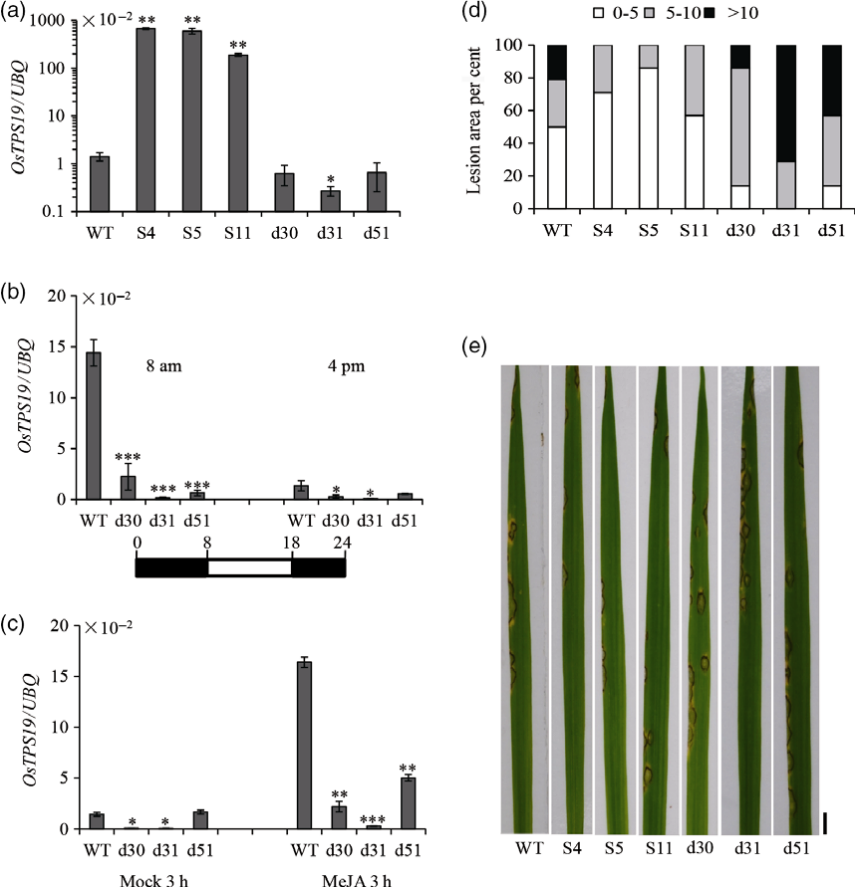
Alternation of *OsTPS* transcription changed resistance against the rice blast fungus. (a) *OsTPS19* transcription in transgenic plants overexpressing *OsTPS19* (S4, S5 and S11) and in the double RNAi lines (d30, d31 and d51) at 4 pm. Expression of *OsTPS19* under the short day condition (10/14‐h light/dark) (b) and MeJA treatment (c) in the double RNAi lines. Samples were collected at designated times. Values presented are the means ± SD of three separate analyses for each RNA template. Asterisks indicate statistically significant differences (Student's *t* test; *P < 0.05, **P < 0.01 and ***P < 0.001). (d) Histograms showing the percentage lesion area categorized into three levels (0–5, 5–10 and higher than 10% of total leaf area). Three‐week‐old transgenic and wild‐type (WT) plants were sprayed with conidial suspensions of *M. oryzae* SZ (10^5^ spores/mL). Lesions were measured on the third leaves of 14 plants per line. (e) Leaves of inoculated plants were photographed 6 days after infection. Bar = 5 mm. Experiments were repeated three times with similar results.

To verify the *in planta* function of *OsTPS19*, transgenic rice plants were generated by means of overexpression or RNAi knock‐down of both *OsTPS19* and *OsTPS20* due to their high similarity. Transgenic rice plants did not exhibit any apparent morphological changes. Three independent overexpression (S4, S5 and S11) and double RNAi (d30, d31 and d51) lines were selected for experimental analyses. First, the expression of *OsTPS19* in transgenic and wild‐type rice plants was measured using quantitative RT‐PCR (qRT‐PCR). *OsTPS19* transcription levels in all three overexpression lines were increased over 100‐fold compared to those in wild‐type plants, while the expression of *OsTPS19* or *OsTPS20* was decreased in double RNAi lines (Figures 2a and S3a). To further evaluate RNAi efficiency, the RNAi lines and wild‐type rice seedlings were subjected to tight diurnal rhythm acclimatization or treated with methyl jasmonate (MeJA), an important signal molecule in plant defence responses. Samples from two time points were collected for the diurnal rhythm treatments when *OsTPS19* expression was at its peak (8 am) and was low in the afternoon (4 pm). Stronger suppression of *OsTPS19* expression was observed in all three RNAi lines of the 8 am samples in relation to the 4 pm samples (Figure 2b). Additionally, the MeJA‐induced *OsTPS19* expressions were remarkably inhibited in the RNAi lines compared with wild‐type plants (Figure 2c). Silencing of *OsTPS20* was also observed in the diurnal rhythms and MeJA treatment (Figure S3b and c).

The availability of transgenic rice plants with altered expression of *OsTPS19* made it possible to determine the biological function of OsTPS19. In this study, we were particularly interested in the possible role of OsTPS19 in defence against the microbial pathogen *M. oryzae*. Six days after the inoculation of *M. oryzae* SZ, the sizes of the lesions on individual leaf blades were quantified. The lesion areas on *OsTPS19* overexpression plants were smaller than those on wild‐type plants. By contrast, the lesions areas on RNAi lines were larger than those on wild‐type plants (Figure 2d and e). Spot inoculation of the pathogen showed similar results (Figure S4), suggesting that OsTPS19 might play a role in the defence reaction of rice. Nevertheless, the contribution of OsTPS20 should not be ignored even though its transcription level is relatively low.

### Transgenic rice plants with altered *OsTPS19* transcription showed altered levels of limonene emission

**Figure 3:**
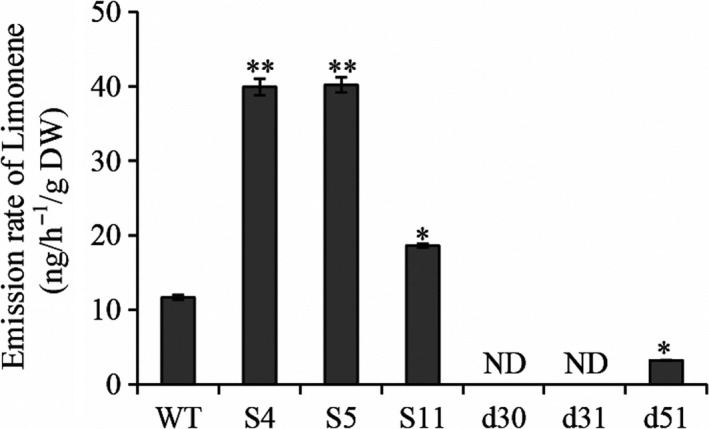
Alternation of *OsTPS* transcription showed altered levels of limonene emission. Limonene emission was measured in three‐week‐old wild‐type (WT) and transgenic seedlings using GC‐MS. ND indicates not detectable due to low amounts. Significant differences between WT and transgenic lines were analysed using Student's *t* test (*P < 0.05 and **P < 0.01).

To understand the mechanisms underlying the *OsTPS19*‐related defence against the fungus, we performed metabolic profiling of the transgenic plants. As OsTPS19 has been reported as a β‐elemene/β‐bisabolene synthase *in vitro* (Taniguchi *et al*., 2014), we measured the headspace of transgenic and wild‐type rice plants to determine the metabolic changes. There were low levels of β‐elemene*,* β‐bisabolene and sabinene emissions among the wild‐type and transgenic lines. Surprisingly, the emission rates of limonene were significantly increased in the overexpression lines but decreased in the RNAi lines (Figure 3). These data indicate that OsTPS19 produces limonene *in planta*.

Limonene is a chiral molecule. Two enantiomers of limonene are known to be produced biologically: (*R*)‐limonene and (*S*)‐limonene. In our previous study, rice plants were shown to release elevated levels of limonene when exposed to fall armyworm larvae (Yuan *et al*., 2008). In this study, volatiles released from rice plants infested with fall armyworm larvae were collected as previously described. The mixture of volatiles was analysed using chiral gas chromatography‐mass spectrometry (GC‐MS). In comparison with authentic standards, the limonene produced by rice plants was determined to be (*S*)‐limonene (Figure S5).

### OsTPS19 has both monoterpene synthase and sesquiterpene synthase activities *in vitro*

To examine the catalytic activities of the OsTPS19 protein, the cDNA of the gene was expressed in *Escherichia coli*, and the recombinant protein was used for the determination of terpene synthase activity. Crude extracts from *E. coli* harbouring only the vector without any *OsTPS* gene inserted were assayed as a negative control.

**Figure 4:**
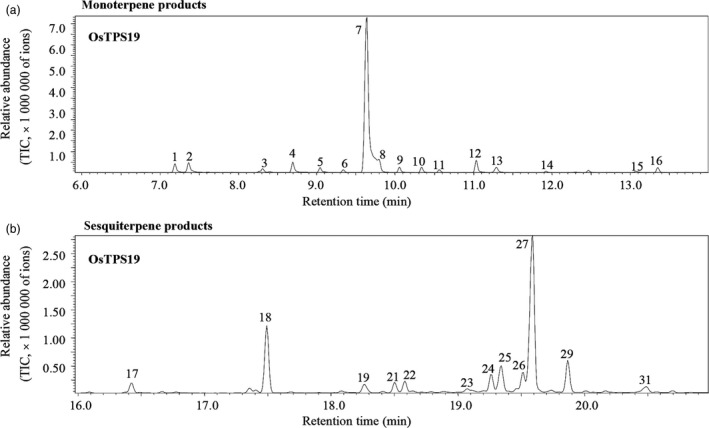
Activities of recombinant OsTPS19. Chromatograms showing the GC‐MS analysis of terpenes produced by recombinant OsTPS19 using geranyl diphosphate (a) and farnesyl diphosphate (b) as substrates. 1, α‐thujene*; 2, α‐pinene*; 3, sabinene*; 4, myrcene*; 5, α‐phellandrene*; 6, α‐terpinene*; 7, limonene*; 8, *cis*‐ocimene; 9, *trans*‐ocimene; 10, γ‐terpinene*; 11, *trans*‐sabinene hydrate*; 12, α‐terpinolene*; 13, *cis*‐sabinene hydrate; 14, neo alloocimene; 15, terpinen‐4‐ol, 16, α‐terpineol; 17, δ‐elemene; 18, β‐elemene; 19, (*E*)‐α‐bergamotene*; 21, unknown; 22, (*E*)‐β‐farnesene*; 23, γ‐curcumene; 24, unknown; 25, zingiberene; 26, (*E,E*)‐α‐farnesene; 27, β‐bisabolene*; 29, sesquiphellandrene; and 31, nerolidol. Compounds marked with asterisks (*) were identified using authentic standards. All other compounds were tentatively identified by comparison of their mass spectra with the WILEY and NIST mass spec libraries.

OsTPS19 was first assayed with geranyl diphosphate, the substrate for monoterpene synthases. OsTPS19 catalysed the formation of 16 monoterpenes, including α‐thujene, α‐pinene, sabinene, myrcene, α‐phellandrene, α‐terpinene, (*S*)‐limonene, *cis*‐ocimene, *trans*‐ocimene, γ‐terpinene, *trans*‐sabinene hydrate, α‐terpinolene, *cis*‐sabinene hydrate, neo alloocimene, terpinen‐4‐ol, and α‐terpineol hydrate (Figures 4a and S5). However, (*S*)‐limonene was the predominant compound.

OsTPS19 was then assayed for sesquiterpene synthase activities. Using farnesyl diphosphate as a substrate, OsTPS19 catalysed the formation of 12 sesquiterpenes with β‐bisabolene as the most abundant product (Figure 4b).

### OsTPS19 is targeted to plastids

**Figure 5:**
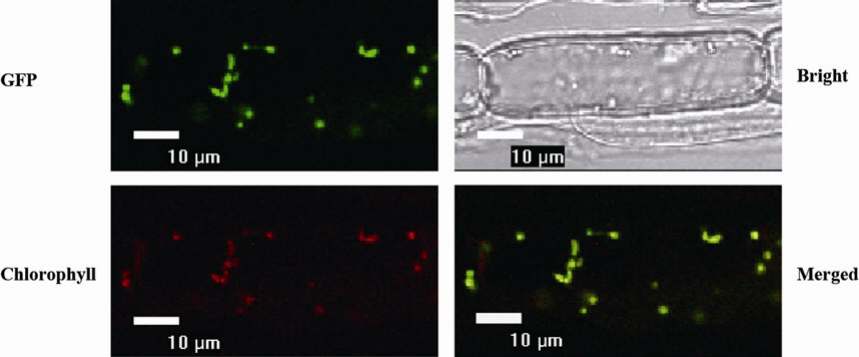
Plastid localization of OsTPS19. The root cells of two‐week‐old *CaMV35S:OsTPS19‐GFP* plants were used for fluorescence signal detection by confocal microscopy. The fluorescence pattern of the subcellular localization of the OsTPS19‐GFP fusion protein (top left panel) completely matched the chloroplast autofluorescence in the merged image (bottom right panel).

*OsTPS19* was constructed to the 5′ end of *GFP,* and the fused gene was controlled by a CaMV35 promoter (*CaMV35S:OsTPS19‐GFP*). Fluorescence of GFP was observed in 2‐week‐old root cells of *CaMV35S:OsTPS19‐GFP* transgenic rice plants of T_1_ progeny (Figure 5) and was colocalized with red autofluorescence of chlorophylls, indicating that the OsTPS19‐GFP protein is targeted to plastids. Additionally, expression of *OsTPS19‐GFP* was performed transiently in leaves of *Nicotiana benthamiana* by agroinfiltration. Colocalization of GFP with red autofluorescence of chlorophylls confirmed the plastidial target of OsTPS19‐GFP (Figure S6).

### Functional variation of OsTPS19 and OsTPS20 is determined by a single amino acid

The high sequence similarity and the close locations of *OsTPS19* and *OsTPS20* suggest that these two genes resulted from a recent gene duplication event. Similar to OsTPS19, OsTPS20 also showed monoterpene synthase and sesquiterpene synthase activities *in vitro* and is targeted to plastids (Figures S7 and S8). In addition to the difference in limonene production, OsTPS19 and OsTPS20 also showed differences in the relative proportions of other minor products. For instance, α‐terpinolene was the second most abundant product of OsTPS19, while OsTPS20 formed *cis*‐sabinene hydrate as the second most abundant product (Figures 4a and S7a).

**Figure 6:**
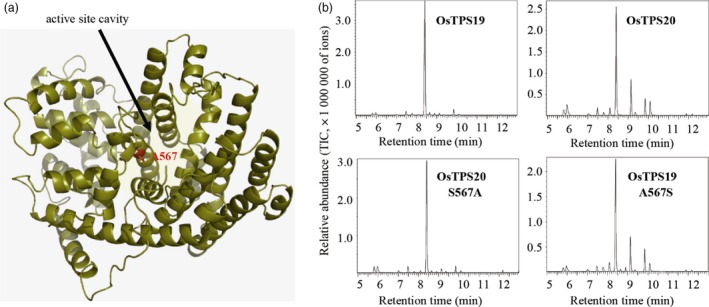
One amino acid is responsible for the different product specificities of OsTPS19 and OsTPS20. (a) Three‐dimensional model of OsTPS19 showing the active site cavity and the position of alanine 567. (b) Enzyme activity of OsTPS19, OsTPS20 and their mutants. The traces of the MS detector are shown for the wild‐type enzymes OsTPS19 and OsTPS20 and their mutants OsTPS19‐A567S and OsTPS20‐S567A.

To understand the structural basis underlying the functional divergence of OsTPS19 and OsTPS20, we generated a homology‐based structural model of OsTPS19 and identified active site residues that differed between OsTPS19 and OsTPS20. Among all the variations between the two proteins, only one residue (alanine 567 in OsTPS19) was localized in the active site cavity (Figure 6a). This amino acid variation was hypothesized to be responsible for the different product specificities of OsTPS19 and OsTPS20.

To test this hypothesis, two mutants, OsTPS19^A567S^ and OsTPS20^S567A^, were created, and the mutant proteins were tested for activity using geranyl diphosphate. The product profile of OsTPS19^A567S^ was highly similar to that of the native OsTPS20, while the product profile of OsTPS20^S567A^ was highly similar to that of the native OsTPS19 (Figure 6b). Thus, the functional variation between OsTPS19 and OsTPS20 was determined by a single amino acid change.

**Figure 7:**
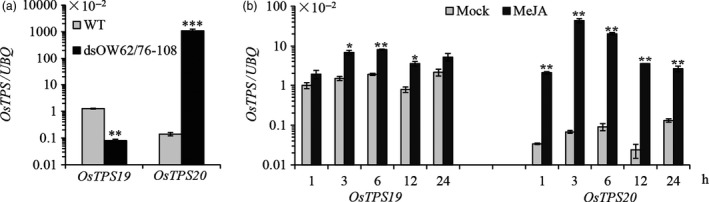
Transcription of *OsTPS19* and *OsTPS20* was differently regulated. (a) Expression of *OsTPS19* and *OsTPS20* in *OsWRKY62* and *OsWRKY76* knock‐down plants (dsOW62/76‐108). (b) Induction of *OsTPS19* and *OsTPS20* transcriptions by foliar treatment of methyl jasmonate (MeJA). The transcription level of each gene was normalized using rice *UBQ*. WT represents wild‐type control plants; mock represents the control treatments. Values presented are the means ± SD of three separate analyses for each RNA template; similar results were obtained from each duplicate. Differences between the mock, and the treatment were analysed using Student's *t* test (*P < 0.05, **P < 0.01 and ***P < 0.001).

To determine whether *OsTPS19* and *OsTPS20* are differentially regulated in the process of subfunctionalization, we examined their transcription in dsOW62/76‐108 plants, which harbour an RNAi construct to knock down both *OsWRKY62* and *OsWRKY76* and show high resistance to *M. oryzae* and bacterial blight pathogen *Xanthomonas oryzae* pv. *oryzae* (*Xoo*) (Liu *et al*., 2016). Interestingly, the transcription of *OsTPS19* was down‐regulated, whereas the level of *OsTPS20* mRNA was remarkably enhanced (Figure 7a). On the other hand, the induction of *OsTPS20* was higher in comparison with *OsTPS19* in rice leaves treated with MeJA (Figure 7b). Additionally, we noticed that the basal transcription level of *OsTPS19* was several fold higher than that of *OsTPS20* in wild‐type plants. These results suggest that *OsTPS19* and *OsTPS20*, two recently duplicated genes, have evolved to respond differently to environmental stimuli.

### (*S*)‐Limonene has an inhibitory effect on the germination of *M. oryzae* spores

**Figure 8:**
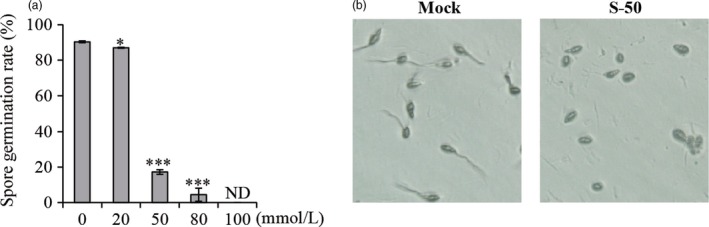
Inhibitory effect of (*S*)‐limonene on the spore germination of *M. oryzae*. An aliquot of 0.1 mL of *M. oryzae* spore suspension (5 × 10^5^ spores/mL) was placed on separate water agar plates that contained various concentrations of (*S*)‐(‐)‐limonene (0, 20, 50, 80 and 100 mmol/L). Each concentration had three repeats. Approximately 200 spores were counted, and the spore germination percentage was calculated (a) and photographed (b) after 5 h of incubation at 28°C. ND indicates not detectable due to no spore germination. S‐50 represents the treatment, in the presence of 50 mmol/L (*S*)‐(‐)‐limonene. Asterisks show statistically differences between the mock and treatment (Student's *t* test; *P < 0.05 and ***P < 0.001).

To assess the effect of (*S*)‐limonene on the pathogen, the germination of *M. oryzae* spores was examined at different concentrations of (*S*)‐limonene. Spore germination was decreased by treatment with (*S*)‐limonene and was even completely inhibited under increased (*S*)‐limonene concentrations (Figure 8). The results imply that (*S*)‐limonene might have a direct role in the suppression of fungal infection.

## Discussion

### OsTPS19 is a rice (*S*)‐limonene synthase gene *in planta*

Among numerous metabolites that may prevent pathogen invasion by forming chemical barriers, terpenoids are the major contributors to the chemical arsenal of plants and play roles in pollinator attraction and signalling to other plants (Dudareva *et al*., 2006). A number of TPSs from a variety of plant species have been well studied, and some of them have been applied for metabolic manipulation (Aharoni *et al*., 2006; Yu and Utsumi, 2009). Sequence comparison indicates that OsTPS19 is identical to AK071447, which has recently been reported as a sesquiterpene synthase in rice (Taniguchi *et al*., 2014). Jasmonic acid treatment of rice plants resulted in a significant up‐regulation of AK071447 expression, correlating with enhanced levels of β‐elemene, one of the *in vitro* sesquiterpene products of recombinant AK071447 (Taniguchi *et al*., 2014). Here, we found that the *OsTPS20* gene was induced more than *OsTPS19* by MeJA treatment using gene‐specific primers (Figure 7b). In addition, OsTPS19 acts as a monoterpene synthase *in planta* (Figure 3). The emission level of limonene was increased less than 10‐fold in transgenic rice plants, likely due to photosynthetic limitations (Wang *et al*., 2016). OsTPS19 was reported to be localized in the cytosol or other organelles other than plastids (Taniguchi *et al*., 2014). However, in our study, for OsTPS19 with almost identical N‐terminal sequences, the subcellular localization was confirmed to be plastidial based on the root cells of stable transformants (Figure 5). Further, the level of limonene, not β‐elemene, increased in overexpression rice plants. The fact that β‐elemene was induced after JA treatment (Taniguchi *et al*., 2014) could be explained by the presence and up‐regulation of a distinct β‐elemene synthase gene. Additional support that OsTPS19 functions as a monoterpene synthase is based on the functional characterization of OsTPS20, which has been shown to also function as an (*S*)‐limonene synthase (Lee *et al*., 2015). OsTPS20 and OsTPS19 are close homologs and are hypothesized to be derived from a relatively recent gene duplication event. It is interesting that OsTPS20 was previously shown to have no sesquiterpene synthase activity (Lee *et al*., 2015). By contrast, our analysis demonstrated that OsTPS20 can catalyse the biosynthesis of sesquiterpenes in *in vitro* assays, similar to OsTPS19.

### The biological functions of OsTPS19

The list of plant terpene synthases with characterized biochemical functions has grown rapidly. Many terpenoids have antimicrobial activities (Ahuja *et al*., 2012). For example, limonene has both antifungal (Tao *et al*., 2014) and bactericidal (Diao *et al*., 2013; Lee *et al*., 2016) activities. *OsTPS19*, identified as a limonene synthase, was characterized to play a role in the defence of rice plants against the fungal pathogen *M. oryzae*. The significant differences in the lesion sizes (Figures 2 and S4) indicate that the *OsTPS19* overexpressors had a stronger resistance to *M. oryzae* than wild‐type rice plants, and the *OsTPSs*‐RNAi lines had a weaker resistance to *M. oryzae*. It is sensible to attribute the different levels of resistance of transgenic and wild‐type rice plants against *M. oryzae* to the different levels of (*S*)‐limonene they produced (Figures 2 and 3). Limonene was one of the volatiles released from pathogen (*M. oryzae* and *Xoo*)‐infected rice seedlings (Lee *et al*., 2016; Obara *et al*., 2002) and be induced dramatically under oxidative abiotic stresses (UV‐B, γ‐rays, and H_2_O_2_) (Lee *et al*., 2015). The spore germination of *M. oryzae* was inhibited by the presence of monoterpene (*S*)‐limonene (Figure 8). Additionally, limonene might be oxidized to limonene hydroperoxide, which acts as an active oxygen species to initiate wide defence responses (Ben‐Yehoshua *et al*., 2008). Some nontarget pathways and products of metabolites (Pasoreck *et al*., 2016) may also be involved in the increased resistance against rice blast fungus in *OsTPS19* overexpression rice plants.

### Evolutionary implications of limonene synthase genes

As described previously, most limonene synthases from angiosperms belong to the TPS‐b subfamily (Chen *et al*., 2011). The identification of the two members of the TPS‐a subfamily in rice encoding limonene synthase raises an interesting question about the evolution of limonene synthase genes in angiosperms. OsTPS19 and OsTPS20 were reported as different types of terpene synthases by Taniguchi *et al*. (2014) and Lee *et al*. (2015, 2016), respectively*. OsTPS20* was found to be induced by oxidative stress or the bacterial pathogen *Xoo* and produces many monoterpenes including (S)‐limonene *in vitro* (Lee *et al*., 2015, 2016).

We hypothesize that the (*S*)‐limonene synthase genes in rice have evolved from an ancestral sesquiterpene synthase gene of the TPS‐a subfamily. Such functional evolution would have involved the change in subcellular localization; that is, the acquisition of a transit peptide by a sesquiterpene synthase would change its biological function to that of a monoterpene synthase. This conclusion is also supported by the fact that in *in vitro* assays, both OsTPS19 and OsTPS20 catalysed the formation of sesquiterpenes from farnesyl diphosphate (Figures 4b and S7b). It remains to be determined whether the lack of the TPS‐b subfamily in rice is the cause or the outcome of the evolution of limonene synthase genes from an ancestral sesquiterpene synthase gene.

After its evolution, the (*S*)‐limonene synthase gene underwent duplication, which resulted in two highly homologous members, that is *OsTPS19* and *OsTPS20*. Their functional divergence occurred at the biochemical level (Figure 6). The mechanism responsible for their functional divergence is the same as that observed previously (for example, Zhuang *et al*., 2012): the change in one or a few key amino acid residues in the active site cavity alters the product specificity. It is equally interesting to observe that functional divergence of *OsTPS19* and *OsTPS20* also occurred at the gene expression level: *OsTPS19* is constitutively expressed and induced by the rice blast fungus, while *OsTPS20* is highly induced by MeJA. The transcription level of *OsTPS19* was increased in the *OsWRKY89* overexpression line but decreased in the *OsWRKY62/76* knock‐down line. By contrast, the expression level of *OsTPS20* was increased in the *OsWRKY62/76* knock‐down line (Figure 7). However, whether the different patterns of *OsTPS19* and *OsTPS20* transcripts in the WRKY transgenic plants resulted from the direct regulation of the transcription factors or are the consequence of endogenous phytohormone changes needs to be clarified. Nevertheless, the *OsTPS19* and *OsTPS20* genes diverged in response to stresses over a wide spatiotemporal range.

## Experimental procedures

### Plant growth and treatments

Rice seeds of wild‐type (*Oryza sativa* L. Zhonghua 17, ZH17 or Xiushui 11, XS11) and transgenic progenies were germinated, and the seedlings were grown in vermiculite at approximately 28°C under a 14/10‐h (light/dark) photoperiod in a greenhouse. For circadian rhythm treatment, a short day condition of 10/14‐h (light/dark) cycle was used (Cai *et al*., 2014). For MeJA treatment, three‐week‐old rice seedlings were treated with 100 μm MeJA (dissolved in 10 mm 4‐morpholine ethanosulphonic acid buffer, pH 5.6) by foliar spray. Treated leaves were sampled at 1, 3, 6, 12 and 24 h after treatment for RNA isolation.

### Terpene synthase enzyme assays

Full‐length cDNAs for *OsTPS19* and *OsTPS20* were cloned using RT‐PCR. The primers used were 5′‐atgtcaacttccatccctctc‐3′ and 5′‐ctaaagggtgacaggattcac‐3′ (reverse, TPS19r) for *OsTPS19* and 5′‐atgtctacttccatccctctc‐3′ and 5′‐ctagatggggacaggattcac‐3′ (reverse, TPS20r) for *OsTPS20*. After cloning the full‐length cDNAs, forward primers of 5′‐atgcgacaaagcagtgcgcatc‐3′ and 5′‐atgcgacaaagcaatgcgcatc‐3′ paired with TPS19r and TPS20r, respectively, were used to amplify the truncated forms of OsTPS19 and OsTPS20. Expression of OsTPS19 and OsTPS20 in *E. coli* and *in vitro* terpene synthase enzyme assays using farnesyl diphosphate and geranyl diphosphate as substrates were performed as previously described (Yuan *et al*., 2008).

### Generation of transgenic plants

*OsTPS19* was amplified using the forward primer 5′‐tattggatcccaagggaaatatactagtatg‐3′ and the reverse primer 5′‐tgttggtacctcgagttctaaagggtgacaggat‐3′, and the PCR product was ligated into a T‐vector (pMD‐*OsTPS19*). The coding region of *OsTPS19* was obtained by enzyme digestion and was cloned into a modified pCambia1301 vector in which *OsTPS19* is under the control of a maize *ubiquitin* promoter (Wang *et al*., 2007). To knock down *OsTPS19*/*20* transcription, a fragment of the *OsTPS19* gene, which was highly similar to *OsTPS20,* was obtained by PCR amplification using the primer pair 5′‐aggaggatccgcaaacacagtagagtgct‐3′ (forward) and 5′‐tgttggtacctcgagttctaaagggtgacaggat‐3′ (reverse). The RNAi construct was placed under the control of the cauliflower mosaic virus promoter (CaMV35S).

For the determination of OsTPS19 subcellular localization, the chimeric gene of *OsTPS19* and the green fluorescent protein (GFP) gene were used to generate the *CaMV35S:OsTPS19‐GFP* construct.

Transgenic plants were obtained by the *Agrobacterium*‐mediated transformation method using Zhonghua 17 as the donor. For each construct, more than 10 independent transgenic lines were screened for hygromycin resistance, and the antibiotic resistant lines were used for experiments.

To detect the subcellular localization of OsTPS20, the cDNA of *OsTPS20* was amplified using PCR with two primers: 5′‐gctagcatgtctacttccat‐3′ (forward), 5′‐cgcggatccgatggggacaggatt‐3′ (reverse). The verified PCR product was cloned into the vector pAN581 to generate a C‐terminal fusion of OsTPS20 to YFP.

### Headspace collection of rice plants and volatile analysis using GC‐MS

The collection of volatiles emitted from 3‐week‐old rice plants was performed using a previously described open headspace system (Sun *et al*., 2017). In brief, volatiles were collected for 24 h on 50 mg of 60/80 mesh Tenax‐TA (Shanghai ANPEL Scientific Instrument Company, Shanghai, China). The collected volatiles were extracted with 300 μL of HPLC‐grade hexane (Fisher Scientific, New Jersey), to which 25.95 ng ethyl decanoate (Sigma‐Aldrich, Oakville, ON) was added as an internal standard. One microlitre of each sample was analysed using a Shimadzu GC‐MS (GC‐MS‐QP2010 SE, Japan) on an Rxi‐5Sil MS column (30 m × 0.250 mm×0.25 μm, Restek, Bad Homburg, Germany). The GC oven temperature program was 40°C for 1 min followed by an increase to 130°C at a rate of 4°C/min (5‐min hold) and then to 250°C at a rate of 10°C/min (5‐min hold). Chiral GC‐MS analysis of limonene was performed using an Rt^™^‐β DEXsm column (Restek, Bad Homburg, Germany) and a temperature program of 40°C (1‐min hold) followed by an increase to 100°C at a rate of 1°C/min and then to 240°C at a rate of 10°C/min (2‐min hold). The flow rate of the carrier gas (helium) was 1 mL/min. Limonene standards were obtained from Sigma.

### Subcellular localization of OsTPS19 and OsTPS20

Localization of OsTPS19 was analysed using *CaMV35S:OsTPS19‐GFP* transgenic rice seedlings. The seeds of the transgenic progenies were germinated on solid half‐strength MS medium for 2 weeks at 28°C under a 14/10‐h light/dark cycle. The cells of 2‐week‐old rice roots were examined using a confocal laser scanning microscope (Eclipse TE2000, Nikon, Beijing, China). For transient expression, the *CaMV35S:OsTPS19‐GFP* plasmid was introduced into the leaves of 4‐week‐old *N. benthamiana* by agroinfiltration. Fluorescence was visualized using the confocal laser scanning microscope.

The plasmid coding for the OsTPS20‐YFP fusion was co‐introduced together with the plastid‐targeted plasmid (pt‐r), which codes for a plastid‐targeted variant of mCherry, into onion epidermal cells by PDS‐1000 biolistic particle delivery system (Bio‐Rad, Hercules, CA). Fluorescence in the epidermal cells was examined 16 h postbombardment using fluorescence microscopy on a Zeiss Axiovert 200M (http://www.zeiss.com) with a 20 ×  objective and appropriate filters (Chroma set 69308; http://www.chroma.com).

### Real‐time quantitative RT‐PCR

DNase‐treated RNA (2 μg) was reverse‐transcribed for analysis of gene expression. Quantitative RT‐PCR (qRT‐PCR) was performed in a 15‐μL reaction volume using SYBR Green dye. The level of rice *ubiquitin* gene (*UBQ*) expression was used to normalize the expression of other genes. Primer sequences used for the qRT‐PCR analysis were 5′‐gcttgctcttcaactcttggga‐3′ (forward) and 5′‐aagattggtcagctttttagtgtctc‐3′ (reverse) for *OsWRKY89,* 5′‐gagtgctatatcaatgagcac‐3′ (forward) and 5′‐acagattcccaacatttccc‐3′ (reverse) for *OsTPS19*, 5′‐cactgtagagtgctatatg‐3′ (forward) and 5′‐caaacagatccccaacaatc‐3′ (reverse) for *OsTPS20*, and 5′‐gtggtggccagtaagtcctc‐3′ (forward) and 5′‐ggacacaatgattagggatca‐3′ (reverse) for *UBQ*.

### Pathogen inoculation

To test disease resistance capability, the wild‐type plants (ZH17) and three‐week‐old transgenic lines were inoculated with the spore solution (10^5^ conidia per millilitre containing 0.02% silwet‐L77) by spraying or spot inoculation as described previously (Fujisaki *et al*., 2015; Wang *et al*., 2007). The area of lesions formed 6 days postinoculation was measured using an image analysis method. For spot inoculation, the vertical length of disease lesions was measured 6 days after inoculation.

### Spore germination assay

An aliquot of 0.1 mL of *M. oryzae* spore suspension (5 × 10^5^ spores/mL) was placed on separate water agar plates containing various concentrations of (*S*)‐(‐)‐limonene. Each concentration had three repeats. Approximately 200 spores were counted, and the spore germination percentage was calculated after 5 h of incubation at 28°C.

### Homology‐based structural modelling

The Swiss‐Model Server (http://www.expasy.org; Schwede *et al*., 2003) was used to generate three‐dimensional structural models of OsTPS19 and OsTPS20. For modelling purposes, the protein sequences of OsTPS19 and OsTPS20 were fitted to the crystal structure of the tobacco 5‐*epi*‐aristolochene synthase mutant C440W (Starks *et al*., 1997; PDB ID, 1HXCA). The quality of the models was assessed using the program ProSA‐web (Wiederstein and Sippl, 2007) as well as the assessment tools provided by the Swiss‐Model Server. The program PyMOL (http://www.pymol.org) was used for visualization of the resulting structural models.

### Site‐directed mutagenesis

The primers used for generating OsTPS19 A567S and OsTPS20 S567A were 5′‐ttttgaacttggccgtagcagtgccattcttttacgat‐3′ and 5′‐tcgtaaaagaatggcactgctacggccaagttcaaaa‐3′, respectively. After confirmation by sequencing, the mutant proteins were expressed in *E. coli*. Terpene synthase activity assays were performed as described in the previous subsection.

## Author contributions

X.C., H.C. J.S.Y, Z.G. and F.C. designed experiments; X.C., H.C. J.S.Y., T.G.K., Z.L.,Y.C., Y.G., X. Z., X. C., Y‐J, Z, J.F. and A.N. conducted experiments and analysed the data; X. C., H.C., Z.G. and F.C. wrote the manuscript.

## Conflict of interest

The authors declare that they have no competing interests.

## Supporting information


**Figure S1** Transcription level of *OsWRKY89*.
**Figure S2** Expression of *OsTPS19* was related to diurnal rhythms.
**Figure S3** Suppression of *OsTPS20* transcription in the double RNAi plants.
**Figure S4** Overexpression of *OsTPS19* enhanced resistance against rice blast fungus through spot inoculation.
**Figure S5** Chiral GC‐MS analysis of limonene emitted from rice seedlings.
**Figure S6** Plastid localization of OsTPS19 in tobacco leaf.
**Figure S7** Recombinant OsTPS20 exhibited monoterpene and sesquiterpene synthase activities.
**Figure S8** Plastid localization of OsTPS20.
