# Supplementary material for: The rice terpene synthase gene OsTPS19 functions as an (S)‐limonene synthase in planta, and its overexpression leads to enhanced resistance to the blast fungus Magnaporthe oryzae
Source: Plant Biotechnol J. 2018 Apr 6;16(10):1778–87. doi: 10.1111/pbi.12914 (PMC6131416; doi:10.1111/pbi.12914)
Supplement: Supplementary file 1 — Figure S1 Transcription level of OsWRKY89. Figure S2 Expression of OsTPS19 was related to diurnal rhythms. Figure S3 Suppression of OsTPS20 transcription in the double RNAi plants. Figure S4 Overexpression of OsTPS19 enhanced resistance against rice blast fungus through spot inoculation. Figure S5 Chiral GC‐MS analysis of limonene emitted from rice seedlings. Figure S6 Plastid localization of OsTPS19 in tobacco leaf. Figure S7 Recombinant OsTPS20 exhibited monoterpene and sesquiterpene synthase activities. Figure S8 Plastid localization of OsTPS20. [file PBI-16-1778-s001.doc]

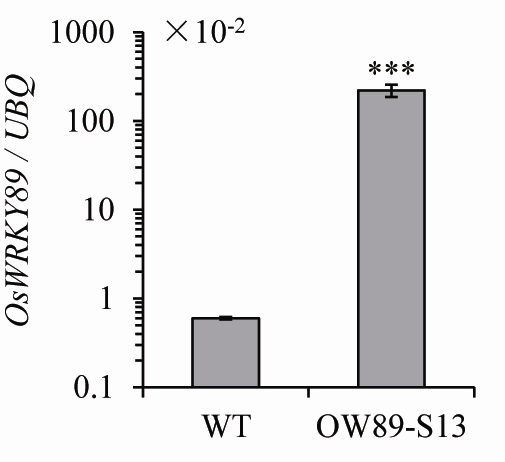


**Supplemental Figure S1 Transcription level of *OsWRKY89*.**

Expression of *OsWRKY89* was determined by qRT-PCR. Total mRNAs from *OsWRKY89* overexpressor (OW89-S13) and wild type (WT) were reverse-transcribed and then used as templates for PCR. Rice *UBQ* gene was used as a standard. Values were means ± SD of three separate analyses for each RNA template; similar results were obtained from each duplicate. Asterisks indicate statistically significant differences (Student’s *t* test; *** P< 0.001).


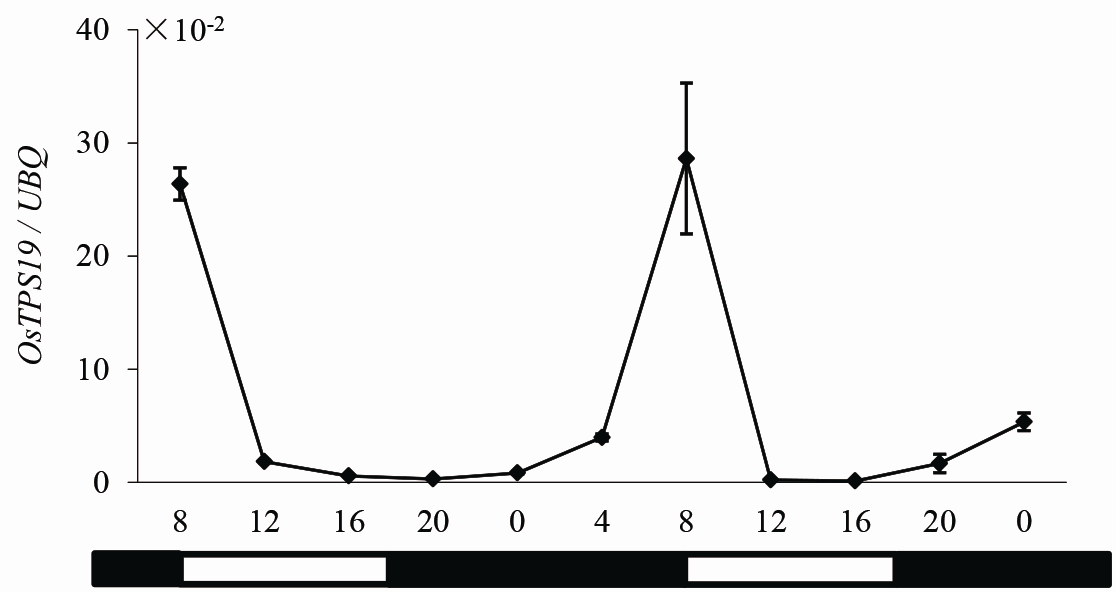


**Supplemental Figure S2 Expression of *OsTPS19* was related to diurnal rhythms.**

Diurnal expression patterns of *OsTPS19* in Zhonghua 11 rice plants under the short day condition (10/14 h light/dark) by qRT-PCR analysis. The expression levels are relative to rice *UBQ* mRNA. Values presented are the means ± SD of two independent experiments.


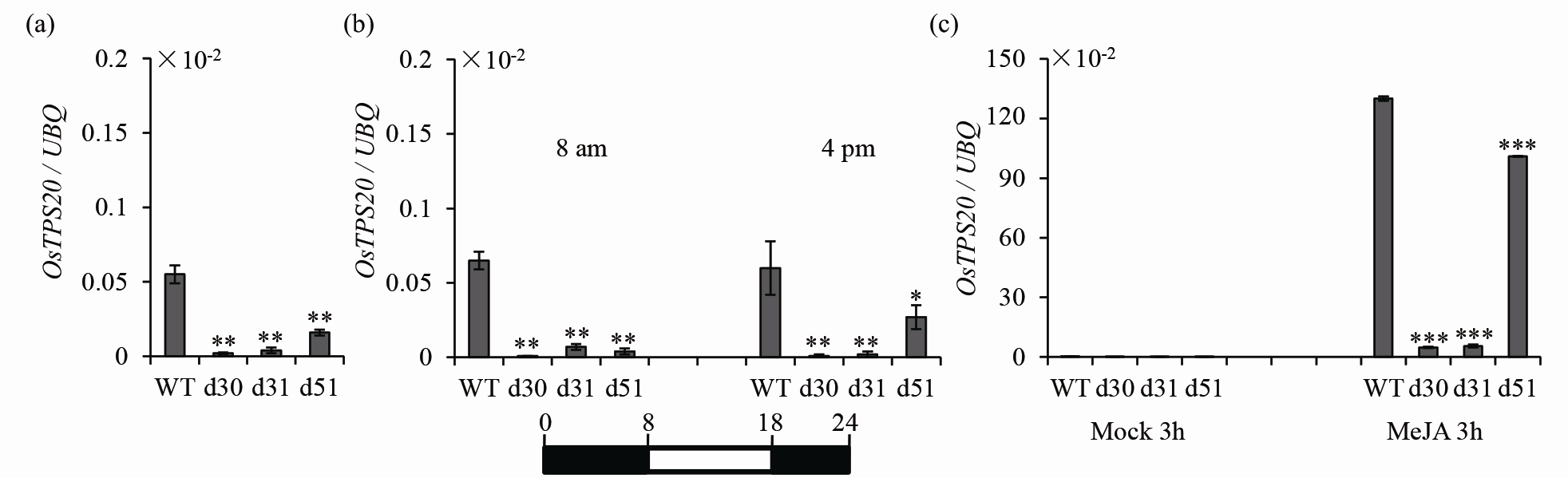


**Supplemental Figure S3 Suppression of *OsTPS20* transcription in the double RNAi plants.**

(a) *OsTPS20* transcription in the double RNAi lines (d30, d31 and d51) at 4 pm. Expression of *OsTPS20* under the short day condition (10/14 h light/dark) (b) and MeJA treatment (c) in the double RNAi lines (d30, d31 and d51) and wild-type plants (WT). Samples were collected at designated time points. Transcription levels were normalized with rice *UBQ* gene. Mock represents the control treatment. Values presented are the means ± SD of three separate analyses for each RNA template. Asterisks indicate statistically significant differences (Student’s t test; * P < 0.05, ** P< 0.01 and *** P< 0.001).


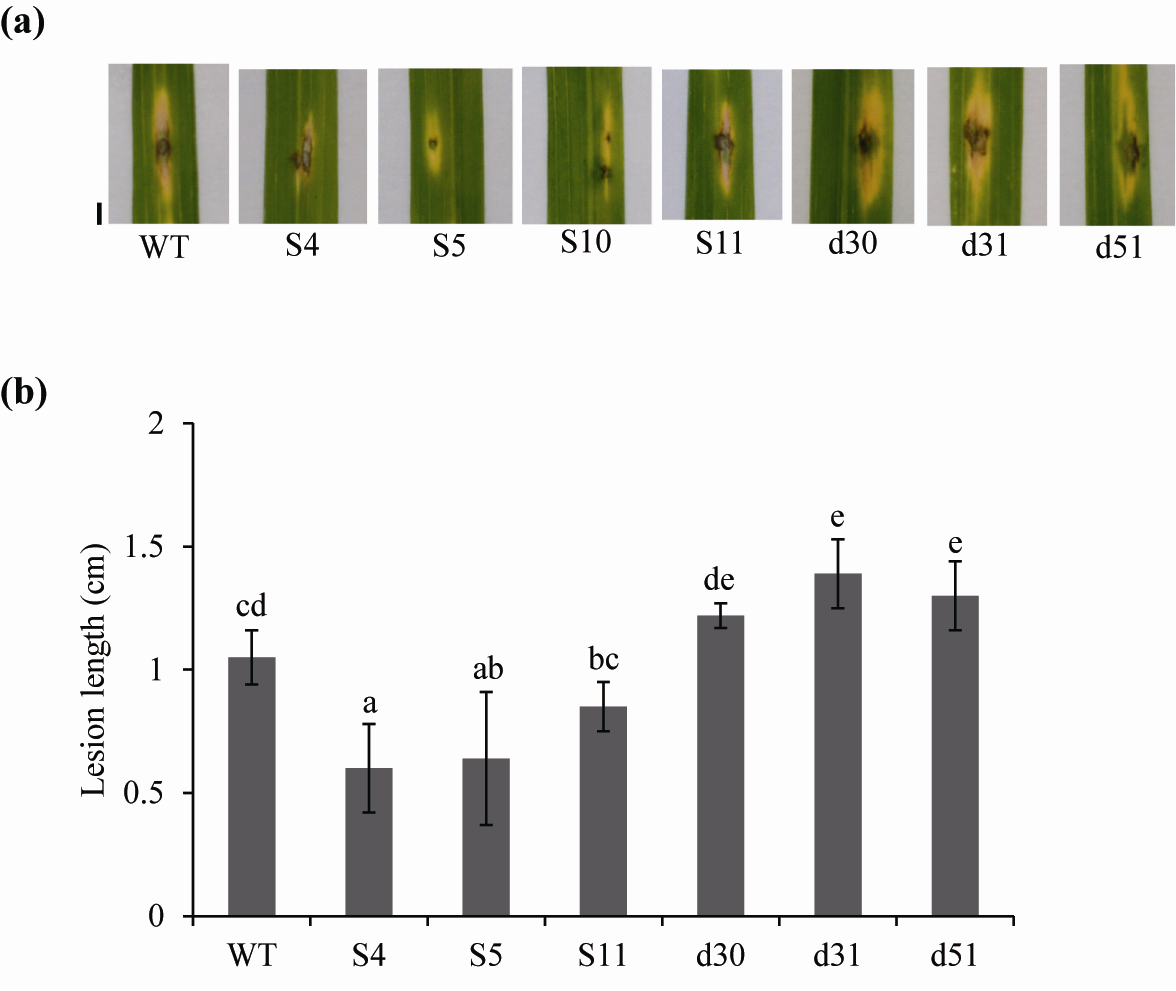


**Supplemental Figure S4 Overexpression of *OsTPS19* enhanced resistance against rice blast fungus through spot inoculation.**

(a) Detached rice leaves were inoculated with *M. oryzae* SZ (10^5^ spores / mL). Leaves were photographed 6 days post the infection. Bar = 2 mm. (b) Lesion lengths are averages of 10 inoculated sections. Values marked with different letters indicate significant differences as analyzed by the SAS software (Duncan’s multiple range test, α = 0.05).


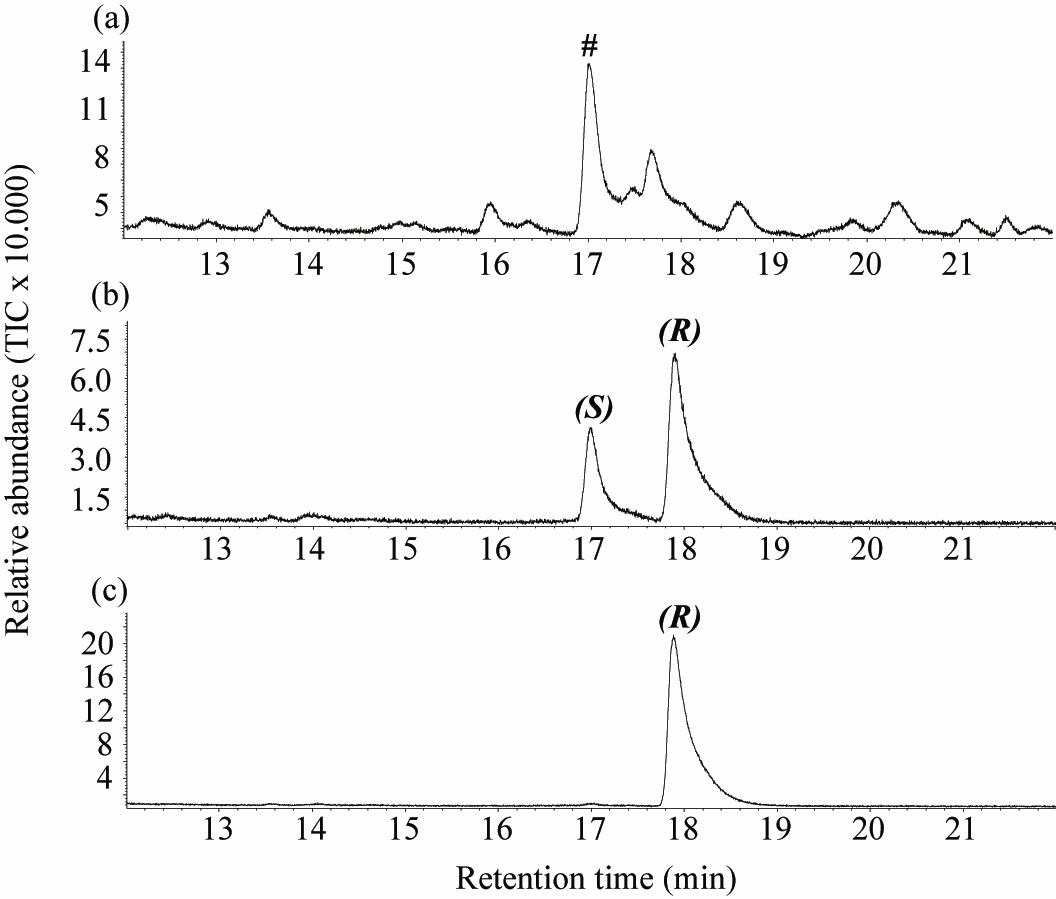


**Supplemental Figure S5 Chiral GC-MS analysis of limonene emitted from rice seedlings.**

(a) GC chromatogram of volatiles emitted from insect-treated rice plants. The peak labelled with “#” depicts limonene. (b) GC chromatogram of a mixture of (*S*)-limonene (*S*) and (*R*)-limonene (*R*) authentic standards. (**c**) GC chromatogram of authentic standard for (*R*)-limonene.


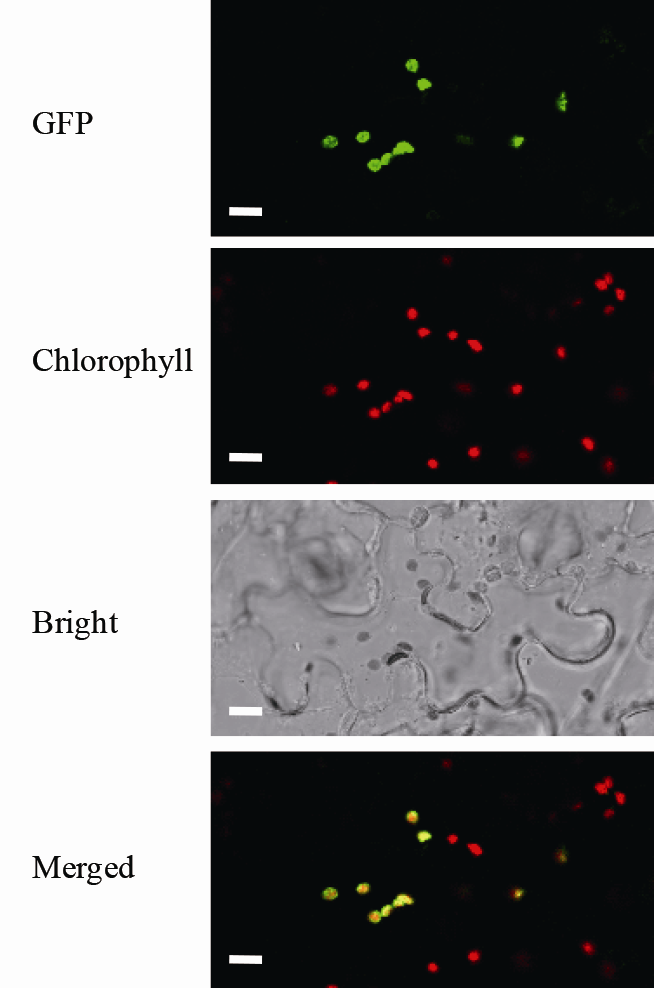


**Supplemental Figure S6 Plastid localization of OsTPS19 in tobacco leaf.**

*CaMV35S:OsTPS19-GFP* plasmid was introduced into the leaves of 4-week-old *N. benthamiana* by agroinfiltrations. Fluorescence was visualized with the confocal laser scanning microscope. The fluorescence pattern of the subcellular localization of the OsTPS19-GFP fusion protein (top panel) completely matched the chloroplast autofluorescence in the merged image (bottom panel). Bar =10 μm.


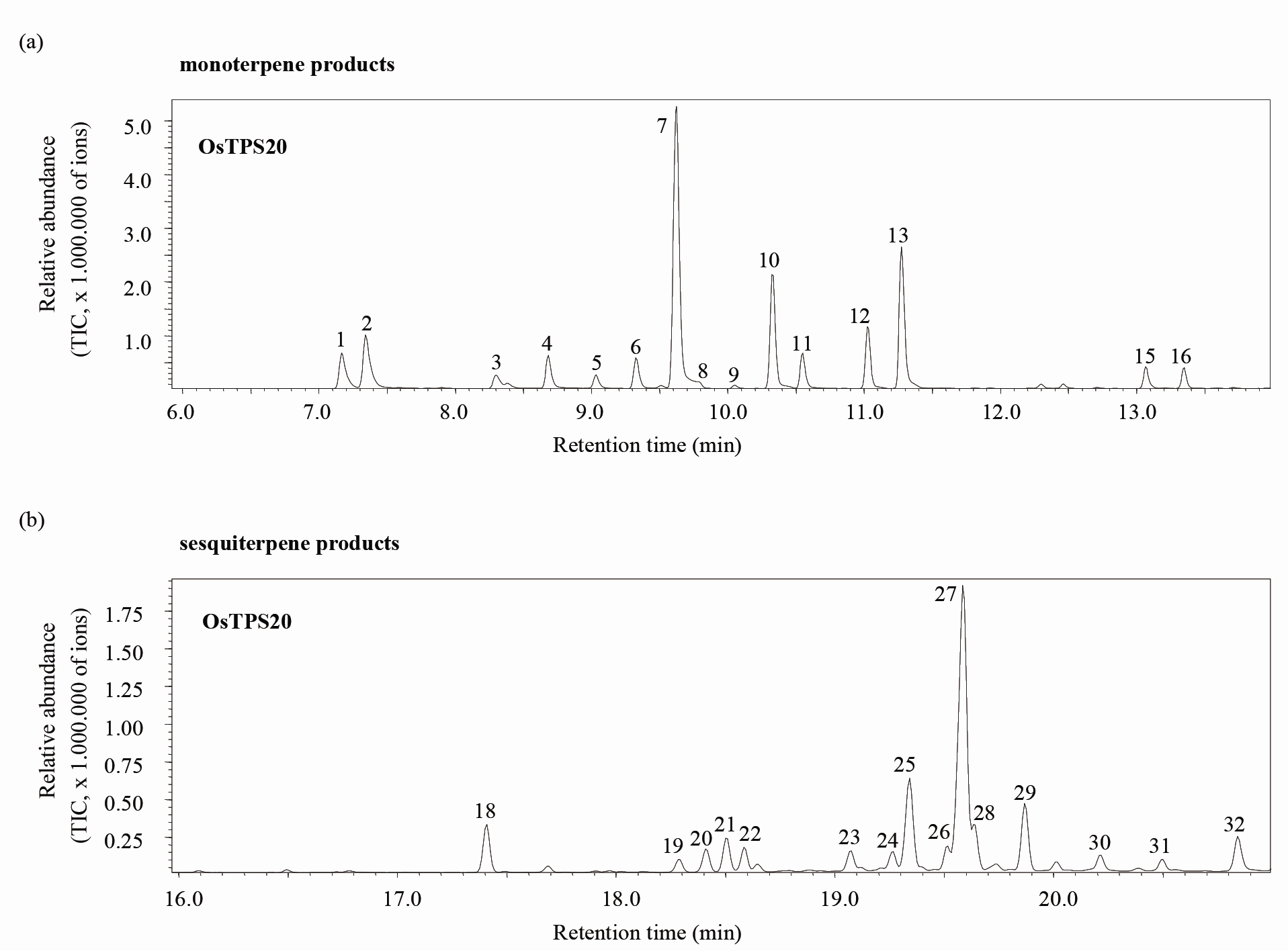


**Supplemental Figure S7 Recombinant OsTPS20 exhibited monoterpene and sesquiterpene synthase activities.**

Chromatograms showing the GC-MS analysis of terpenes produced by recombinant OsTPS20 using geranyl diphosphate (a) and farnesyl diphosphate (b) as substrate.1, α-thujene*; 2, α-pinene*; 3, sabinene*; 4, myrcene*; 5,α-phellandrene*; 6,α-terpinene*; 7, limonene*; 8, *cis*-ocimene; 9, *trans*-ocimene; 10, γ-terpinene*; 11, *trans*-sabinene hydrate*; 12, α-terpinolene*; 13, *cis*-sabinene hydrate; 15, terpinen-4-ol, 16, α-terpineol;18, β-elemene; 19, (*E*)-α-bergamotene*; 20, sesquisabinene A*; 21, unknown; 22, (*E*)-β-farnesene*; 23, γ-curcumene; 24, unknown; 25, zingiberene; 26, (*E,E*)-α-farnesene; 27, β-bisabolene*; 28, β-curcumene; 29, sesquiphellandrene; 30, sesquisabinene hydrate 1; 31, nerolidol and 32, sesquisabinene hydrate 2. Compounds marked with asterisks (*) were identified using authentic standards. All other compounds were tentatively identified by comparison of their mass spectra with the WILEY and NIST mass spec libraries.


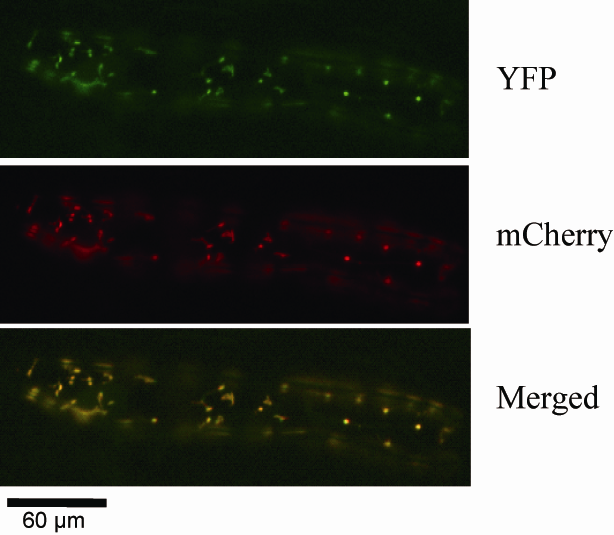


**Supplemental Figure S8 Plastid localization of OsTPS20.**

Colocalization of OsTPS20 with plastid marker in onion epidermal cells. YFP (yellow fluorescence protein) fusion to OsTPS20 was shown in green. mCherry plastid marker was shown in red.
